# Manufacturing of Closed Impeller for Mechanically Pump Fluid Loop Systems Using Selective Laser Melting Additive Manufacturing Technology

**DOI:** 10.3390/ma14205908

**Published:** 2021-10-09

**Authors:** Alexandra Adiaconitei, Ionut Sebastian Vintila, Radu Mihalache, Alexandru Paraschiv, Tiberius Florian Frigioescu, Ionut Florian Popa, Laurent Pambaguian

**Affiliations:** 1 Satellites and Space Equipment Department, Romanian Research and Development Institute for Gas Turbines (COMOTI), 061126 Bucharest, Romania; sebastian.vintila@comoti.ro (I.S.V.); radu.mihalache@comoti.ro (R.M.); ionut.popa@comoti.ro (I.F.P.); 2 Gas Turbine Special Equipment, Physics and Mechanical Testing Laboratory, Romanian Research and Development Institute for Gas Turbines (COMOTI), 061126 Bucharest, Romania; alexandru.paraschiv@comoti.ro (A.P.); tiberius.frigioescu@comoti.ro (T.F.F.); 3 European Space Research and Technology Centre (ESA-ESTEC), Mechanical Department, European Space Agency, 2200 AG Noordwijk, The Netherlands; laurent.pambaguian@esa.int

**Keywords:** additive manufacturing, selective laser melting, closed impeller, MPFL pumps, balancing, non-destructive testing

## Abstract

In the space industry, the market demand for high-pressure mechanically pumped fluid loop (MPFL) systems has increased the interest for integrating advanced technologies in the manufacturing process of critical components with complex geometries. The conventional manufacturing process of a closed impeller encounters different technical challenges, but using additive manufacturing (AM) technology, the small component is printed, fulfilling the quality requirements. This paper presents the Laser Powder Bed Fusion (LPBF) process of a closed impeller designed for a centrifugal pump integrated in an MPFL system with the objective of defining a complete manufacturing process. A set of three closed impellers was manufactured, and each closed impeller was subjected to dimensional accuracy analysis, before and after applying an iterative finishing process for the internal surface area. One of the impellers was validated through non-destructive testing (NDT) activities, and finally, a preliminary balancing was performed for the G2.5 class. The process setup (building orientation and support structure) defined in the current study for a pre-existing geometry of the closed impeller takes full advantages of LPBF technology and represents an important step in the development of complex structural components, increasing the technological readiness level of the AM process for space applications.

## 1. Introduction

Additive manufacturing technology has gained a large amount of interest due to its manufacturing advantages in obtaining components and structures with complex shapes. The use of its applications in the space industry is significantly advancing, for example, the Juno (Jupiter Near-polar Orbiter New Frontiers 2) spacecraft (launched in 2011) was equipped with additively manufactured brackets, and Aerojet Rocketdyne Company (El Segundo, CA, USA) uses this type of manufacturing for LOX/H2 rocket engine injectors and thruster systems for CubeSats and other small satellites [1]. Additive manufacturing has the potential to re-design the space system architectures with a long-term impact to reduce costs and increase performances, taking into consideration design and structural requirements. The competitive environment in the space industry regarding the use of AM is also maintained by the global market dynamics, where the AM industry expanded by 7.5%, reaching near to USD 12.8 billion in 2020 [2]. 

One of the critical technology areas of the NASA Space Technology Roadmap is the Thermal Management Systems, where technology development is needed to enable space exploration and to integrate advanced and additive manufacturing technology. The active thermal systems, where a liquid coolant is circulated in a closed loop under the action of a pump, are known as Fluid Loop Systems, and these are able to maintain the functionality of the spacecraft during the extreme temperature differences, specific for the space environment. The MPFLs present high interest for Europe Large System Integrators (LSI) as a continuous demand for communication satellite platforms that use electric power of around 25 kW [3]. In MPFL, the pump is considered the weakest component and the most likely to fail during operation, and the most used type of centrifugal pump is the one with a canned rotor. The hydraulic capacity and pump performances rely on the impeller, a critical component with a very small and complex shape; therefore, the advantages of AM technology could be used in the manufacturing process in order to achieve a simplified process, mass and costs savings and high performances. Having a low-volume, complex design that formative or subtractive methods are unable to produce, the AM technology fulfils the requirements for manufacturing the closed impeller.

The manufacturing process of the closed impeller using different AM technologies was investigated by Allison et al. [4], and the results showed that the tested Direct Metal Laser Sintering (DMLS) impellers possess acceptable mechanical characteristics, even when some localized material yielding was experienced during speed testing. The hydraulic performance of an AM closed impeller was the major concern of Fernandez’s study [5]. The authors concluded that the inherent roughness of the Fused Deposition Modelling (FDM) process did not limit the head-flow curve results of the pump, and by using a chemical post-treatment, assures a more stable behavior in the high flow operating range of the pump. As the AM technology permits the fabrication of pump impellers with a significantly reduced lead-time compared with conventional processes (casting and machining), Rettberg et al. [6] focused on a new additive manufacturing approach for closed impellers. Sulzer (Winterthur, Switzerland) is developing an impeller manufacturing process, which combines Laser Metal Deposition (LMD) with subtractive 5 Axis CNC Milling. Additionally, Sulzer presented different orientations and support structure designs in order to avoid material deposition in inaccessible areas for a LPBF closed impeller [7]. Consequently, efforts were made by Yanghi et al. [8] towards optimizing the manufacturing process of a closed impeller using Laser Powder Bed Fusion (L-PBF), in order to mitigate the distortions of the part by applying the finite element method (FEM). The validated FEM predicted distortion was used to compensate for distortion at the design stage by numerical reverse engineering, and a new impeller was produced following the same AM and post-machining procedures, resulting in a distortion-compensated impeller with mitigated distortion. 

The current study focused on a specific AM LPBF method for manufacturing a closed impeller out of Inconel 625. Through this process, the part was fabricated by the sequential addition of material. Although it is known as a near-net-shape technique, as the part is directly built based on a computer aided design (CAD) model and no tools are required to initiate the manufacturing process (except the ones required by post-processing), not all technically feasible approaches to additive manufacturing parts are adaptable for space applications. A detailed investigation for space application demanding requirements, such as dimensional accuracy, quality of the surface or material characterization, is required for a better understanding of additive manufacturing processes. Multiple research studies have aimed to perfect and understand the various additive manufacturing techniques, expand the type of materials and parts that are used, and explore the manufacture of a complex, integrated system. Huber et al. [9] described the LPBF manufacturing of an already existing closed impeller design at the lower limit of castability, and the purpose was to find the optimal building direction as well as to design a proper support structure. The results show that LPBF processed prototype closed impeller, obtained through a heuristic and iterative process, fulfilled all geometrical requirements. Thomas [10] developed a set of design rules to achieve more predictable and reliable results regarding LPBF parts. The geometric limitations of LPBF were evaluated through a quantitative cyclic experimental methodology, and an important conclusion was presented regarding the self-supporting surfaces where alternative orientations can eliminate the need for support and can improve the surface quality of the down-facing surfaces. The same subject of surface quality on different sides of Inconel 625 parts was studied by Mumtaz [11], and it was concluded that the parameters that aid a reduction in both top and side surface roughness are achieved through the use of a higher peak power due to the flattening/smoothing of the melt pool surface (due to increased recoil pressure). Additionally, Yang et al. [12] studied the influence of process parameters on the vertical surface roughness of AlSi10Mg parts and concluded that the surface roughness was reduced to 4 μm from 15 μm when a proper linear energy density was used, improving the surface roughness by more than 70%. 

Particular attention is paid to the accuracy of AM parts, and the geometrical accuracy tolerances were found to be ±20–50 μm, even reaching 100 μm [13]; nonetheless, the part-quality may vary due to the nature of layer-by-layer processing. Kamarudin et al. [14] highlighted the dimensional accuracy analysis of the SML benchmark model. Positive deviations of 11.66% (maximum) were identified for the cylinder part and a maximum negative deviation of −3.30% for a rectangular slot. Therefore, the dimensional accuracy may vary depending on the geometry of the printed part and process parameters, as demonstrated by Wang et al. [15]. Overall, the LPBF technology can produce geometrical features, such as sharp corners and cylinders. 

As most alloys (Al-Cu-Mg-Sc-Si, Ni-Cr-based super alloys or Ti6Al-4V) used in the AM field to create complex geometries have been developed for traditional manufacturing processes, such as casting and forging, the performances of the AM part could be severely limited if the influence of the LPBF process on the microstructure and mechanical properties of the material is not properly assessed. In this context, several studies were conducted for a deeper understanding control of the effects that currently limit the fidelity of LPBF as a microstructure, residual stress, micro-roughness and porosity of AM materials. Simonelli et al. [16] investigated the influence of Fe on the microstructural development of Ti-6Al-4V used for LPBF, and Gussone et al. [17] demonstrated the feasibility of Ti-Fe alloys used for LPBF with ultrafine microstructures and mechanical strength for structural application. In [18] and [19], the authors provide a brief overview of alloy design strategies, highlighting the potential for alloys to match to the unique processing conditions encountered during the AM process. In the work performed by Shi et al. [20], the effects of laser beam shape on the temporal evolution of the melt pool geometry were investigated, while Roehling et al. [21] identified different strategies to control microstructures locally and to tailor the mechanical performance of additively manufactured parts.

All the ongoing research activities prove the increasing interest in the AM field not only for the general advantages related to complex shapes, but also for the role of alloys tailored for LPBF.

The activities performed in this study follow a preliminary analysis [22], where an investigation of three different building orientations was conducted (0, 32 and 45°) for an Inconel 625 closed impeller. The impeller orientation on the building plate was selected based on two main criteria. First, the major concern was to avoid as much as possible the deposition of the support structure on the internal surface area. Due to the very small dimension of the impeller (φ 22.2 mm internal diameter), it would have been impossible to remove it. Second, it is well known that an orientation around 45° is best suited for a lower roughness and high dimensional accuracy. Having a self-supporting surface inside of the impeller (the shroud), it was important to achieve the minimum roughness from the printing process. The high roughness of the down-facing surfaces is a common disadvantage of the AM technology. The printed closed impellers were subjected to dimensional accuracy and surface quality evaluations, and it was observed that by increasing the printing angle, a better dimensional stability was obtained for both the exterior regions as well as for the blade surface accuracy. On the suction side of the 32° oriented closed impeller, the deviations were between −0.238 and 0.140 mm. Increasing the printing angle to 45°, the deviations were considerably reduced to the range −0.056 to +0.010 mm. The same observation was applicable for the pressure side of the blades: deviation of the 32°-oriented closed impeller was between −0.153 and +0.204 mm, but for the 45°-oriented part, the deviation was between −0.092 and +0.111 mm. Additionally, post-processing activities were preliminary evaluated; however, the finishing process was not uniform on the entire length of the blade, as the suction sides of each blade remained unfinished. 

Considering the preliminary work mentioned above, the current study proposes an optimized manufacturing process of closed impellers with a geometry that is difficult to achieve through traditional methods (casting or welding), considering LPBF technology. The AM closed impeller was subjected to detailed non-destructive analyses, such as X-ray computer tomography (CT) scans, liquid penetration evaluation, dimensional accuracy, surface finishing and quality analysis. Additionally, the closed impeller was subjected to balancing activities in the G2.5 balancing class, as a preliminary step, into developing an AM rotary component for MPFL systems. The manufacturing process is detailed below, which analyzed from the CAD model to the complete post-processed closed impeller. 

## 2. Materials and Methods

### 2.1. Design Approach

While the level of part complexity that metal printing is able to produce exceeds that of traditional manufacturing techniques, the primary challenge for AM space products is the fulfilment of qualification requirements and the guarantee that all batches of parts have the expected mechanical properties and the same high quality. 

**Figure 1:**
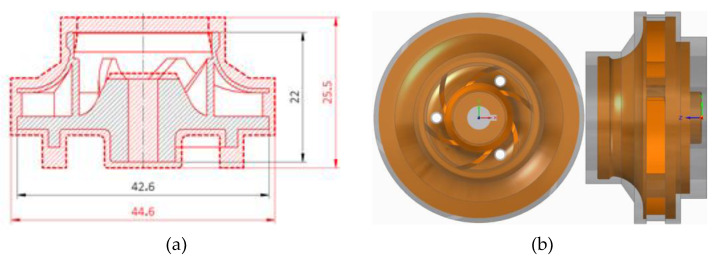
Schematic representation of (**a**) the closed impeller, where black area represents baseline model and red area represents AM model; top and side view of (**b**) the baseline model (orange) with offset material (gray).

The baseline model of the closed impeller and the AM model were designed using Solid Edge (version 2019, Siemens PLM Software, Cologne, Germany), following the AM recommendations and constrains [23,24,25,26,27,28,29]. The closed impeller design for AM does not have any holes (keyway and thrust balancing holes) to prevent the retention of the metal powder and the deposition of the support structure. Additionally, the closed impeller has an offset material on the outer surface for post-processing operations. No additional material was added on the internal surfaces. The outside diameter of the baseline model is 42.6 mm, and the height is 22 mm, but for the AM closed impeller, the outer diameter is 44.6 mm and the height is 25.5 mm, as seen in Figure 1. 

### 2.2. Printing Process Parameters

The closed impellers were manufactured out of Inconel 625 (purchased from LPW Technology Ltd., Runcom, UK), using a Lasertec 30SLM facility (DMG MORI, Bielefeld, Germany) with a building volume of 300 × 300 × 300 mm (L × W × H). The chemical composition of Inconel 625 powder is presented in Table 1 and printing parameters are presented in Table 2. The in-depth material characterization of the Ni-Alloy was performed, and the performances and capabilities of Lasertec 30 SLM (supplier DMG MORI, Bielefeld, Germany) were analyzed in order to define the optimized process parameters to produce high density material. Specimens were manufactured using variable process parameters and were subjected to density and porosity measurements in order to define the most appropriate workspace that generates material with higher relative densities as compared to the theoretical density of the IN 625 alloy. Additionally, the influence of process parameters on the specimen surface roughness and material hardness was assessed. The main conclusion was that for 250 W laser power, 700–800 mm/s scan speed, and layer thicknesses in the range of 30–50 μm, the relative densities achieved are over 99.5%, as highlighted by the authors in [30,31,32]. However, during the manufacturing of the closed impeller and due to the appearance of the adherent dross on the interior side of the impeller, subjected to analysis in [33], the laser power was decreased to 200 W, which was found to be the best corrective measure.

### 2.3. Post-Processing Operations 

The as-printed closed impeller was subjected to heat treatment using an electric air furnace (Nabertherm LH 30/14 GmbH, Lilienthal/Bremen, Germany) that involves stress relief heat treatment (heating with 10 °C/min up to 870 °C, held for 1 h, followed by air cooling) and annealing heat treatment (heating up to 1000 °C, held for 1 h, followed by fast cooling and oil quenching). Post-processing operations were performed for both the interior and exterior surfaces of the closed impeller, in three separate steps. The first step comprises removing support material from the closed impeller, followed by an interior post-processing operation, and finally machining the exterior of the closed impeller to its final dimensions, as defined by the baseline model in Figure 1.

The removal of support material and machining of the exterior surfaces was performed on a conventional lathe turning machine. Abrasive Flow Machining (AFM) was performed on the closed impellers’ interior surface, at Extrude Hone GmbH, Remscheid, Germany, using a VECTOR 6 AFM system, which is ideal for polishing and deburring the internal surface with a small and complex geometry. This technology uses a chemically inactive or non-corrosive media to enhance the roughness and edge conditions. The abrasive particles in the media grind away rather than shear off the unwanted material. Turning operations for the external surface area follow the AFM process as the final machining process of the closed impeller to its final dimensions, as a cost-effective and in-house process.

### 2.4. Verification Plan 

**Figure 2:**
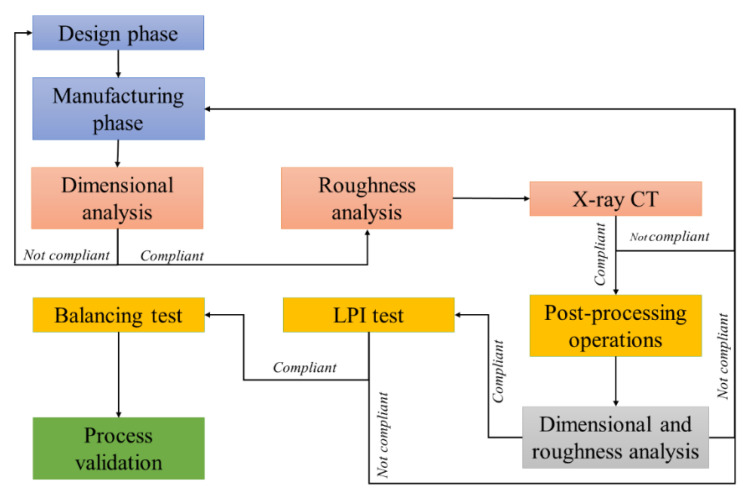
Verification plan—flow chart.

As the AM closed impeller must be free of internal defects, contamination, cracks, lack of fusion or inclusions, and respect the imposed geometrical accuracies and roughness requirements, a verification plan was considered by performing X-ray CT, dye Liquid Penetrant Inspection (LPI), dimensional control and roughness measurements, and the flow-chart is presented in Figure 2.

#### 2.4.1. Non-Destructive Tests

**Figure 3:**
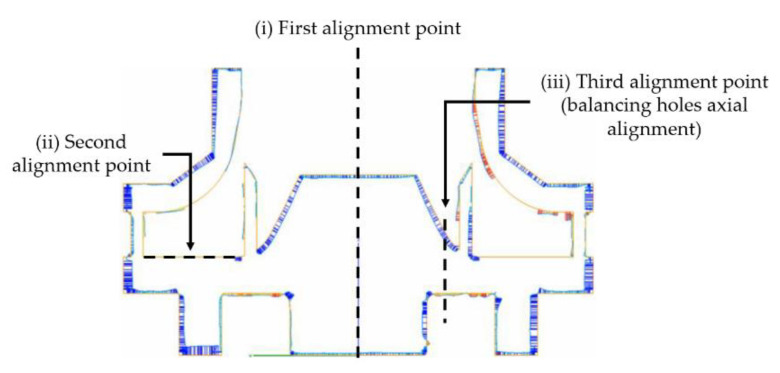
Alignment procedure for the measured model of the closed impeller and the CAD model.

An X-ray CT scan was performed on a Micro CT System (Diondo GmbH, Hattingen, Germany) at Dynamic Instruments (Bucharest, Romania) with a resolution of 20 μm and a dimension of 8 Voxels for investigating defects/porosities (Voxel size of 0.041 mm on all three directions). LPI was performed using MR 71 Cleaner, MR 68 NF Dye Penetrant and MR 70 Developer (MR Chemie GmbH, Unna, Germany) to observe any defects that may appear during or after machining the external surfaces of the AM closed impeller. Roughness was measured using a Mahr Surf PS10 instrument (Mahr GmbH, Gottingen, Germany) before and after post-processing operations for process validation. Length of measurement was considered 0.8 × 10 mm with a 1.0 mm/s speed with respect to impellers dimensions. Dimensional accuracy analysis was performed using a 3D laser surface scanning ATOS Compact Scan 5M machine, integrated with GOM’s software for scanning and inspection with 2 × 5 × 106 pixels and measuring point distance between 0.017 and 0.481 mm. The correlation between the measured model of the closed impeller and the CAD model was performed by means of three-point alignment. The three alignment points were: (i) the closed impeller axis of rotation; (ii) interior top disc surface; (iii) thrust balancing holes axis, as presented in Figure 3. 

#### 2.4.2. Balancing Operations 

The balancing activity is a mandatory step in the development process of this closed impeller in order to ensure a proper operation and the lifetime requirements for an MPFL pump. The balancing procedure was conducted following ISO standard 1940-1: 2003 (E) [34], where due to the small dimensions and mass of the closed impeller, the minimum acceptable class is G2.5. The balancing operations were performed in a single correction plane, with a Passio 5 balancing machine (SCHENCK RoTec GmbH, Darmstadt, Germany) at Aeroteh SA (Bucharest, Romania). The unbalance measurements were conducted at 2200 rot/min. 

## 3. Results

**Figure 4:**
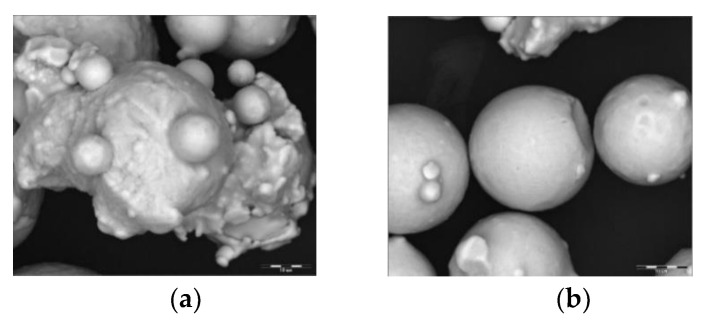
Powder particle shape and morphology: (**a**) particles with satellites joined during solidification; (**b**) smooth, round particles.

The morphology of the virgin IN 625 powder used in the current study presents a typical morphology for gas atomized powder consisting of relatively small particles (size range 10–45 μm), and mainly spherical particles with satellites joined during solidification. Smooth, spherical powder particles were observed as well as elongated particles. Representative SEM images can be observed in Figure 4. 

**Figure 5:**
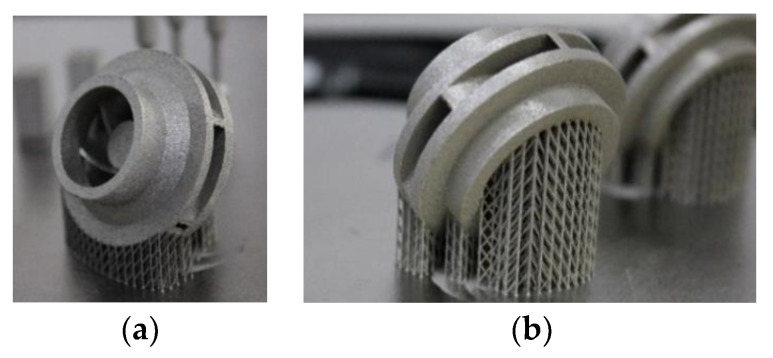
Closed impellers built at B + 60° orientation: (**a**) front view; (**b**) the back view of closed impeller.

**Figure 6:**
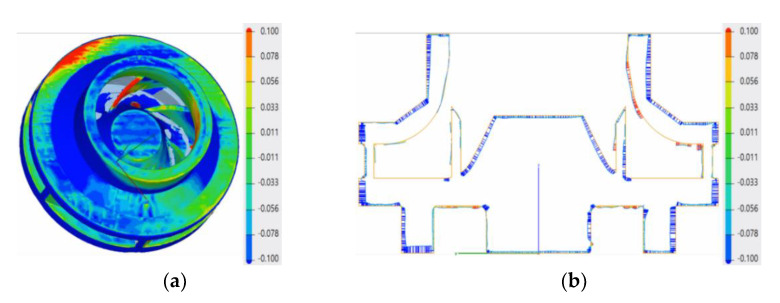
Dimensional evaluation: (**a**) top view; (**b**) section view.

Using the results of the analysis performed by the authors in [33] with respect to an inherent defect of LPBF parts, more precisely, an adhered dross on overhanging structures, it was concluded that the adherent dross can be minimized using the orientation of B + 60° for the closed impeller and a maximum laser power of 200 W. Consequently, a set of three closed impellers at B + 60° orientation were manufactured (Figure 5), for which dimensional evaluation, post-processing operations and a balancing test were performed. The terminology for building orientations is in line with standard terminology for additive manufacturing [35]. The three closed impellers were subjected to heat treatment and the removal of support material before further investigations. Dimensional stability evaluation is presented in Figure 6. The red color on the top of the impeller represents a small area with a high roughness due to the support material still being attached to the shroud. The highest deviation was found on the exterior of the impeller (−0.2 mm), but it can be considered negligible as the offset was set to 1 mm.

Blade positioning was found to have a maximum deviation of ±0.09 mm, showing that the printing process follows the geometrical constraints of ±0.1 mm on the blade positioning and tolerances, with respect to the CAD model.

**Figure 7:**
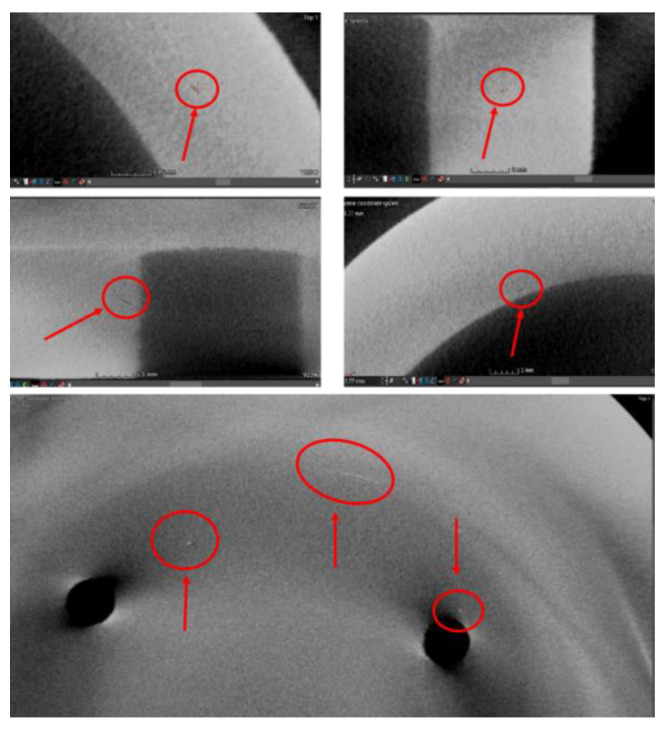
Representative X-ray CT scans for one closed impeller built at B + 60° orientation.

An X-ray CT scan (voxel size of 0.041 mm on all three directions) was performed on all closed impellers to detect any internal defects (pores, lack of fusion, cracks or inclusions), before the post-processing activities. Some representative X-ray CT results are presented in Figure 7 for one of the closed impellers. Small voids in the material were identified, both in depth of the material and on the added offset material. As the added offset material was to be machined, only the defects found internally were analyzed more carefully. The identified defects were measured using the MyVGL software (Volume Graphics GmbH, Heidelberg, Germany), where pores with diameters between 0.04 and 0.09 and superficial cracks with lengths between 0.26 and 0.61 mm were found. However, due to the small size and low volume of the voids identified by the CT scan, it can be concluded that they do not affect the mechanical properties of the closed impeller. This conclusion is also supported by the results of the tensile tests, presented in Table 3, and the obtained relative density (average between 99.4 and 99.5%) and porosity (average between 0.5 and 0.6%), higher than imposed values (min. 99.3% for relative density and max. 0.7% for porosity). Tensile test specimens of 3 mm gauge diameter were machined from 3.5 mm diameter printed coupons. A tensile test was performed on Instron 3369 equipment (Instron, Norwood, MA, USA) with a ±50 kN cell force. The strain rate was set to $\dot{e}$*Lc* = 0.00025 s^−1^ until the detection of yield strength, then the strain rate was changed to $\dot{e}$*Lc* = 0.0067 s^−1^, in accordance with the ISO 68921-1:2009 standard [36].

Based on tensile test results presented in Table 3, it was concluded that the tensile properties of IN 625 specimens built with 200 W laser power and 750 mm/s scanning speed meet the requirements of [37].

**Figure 8:**
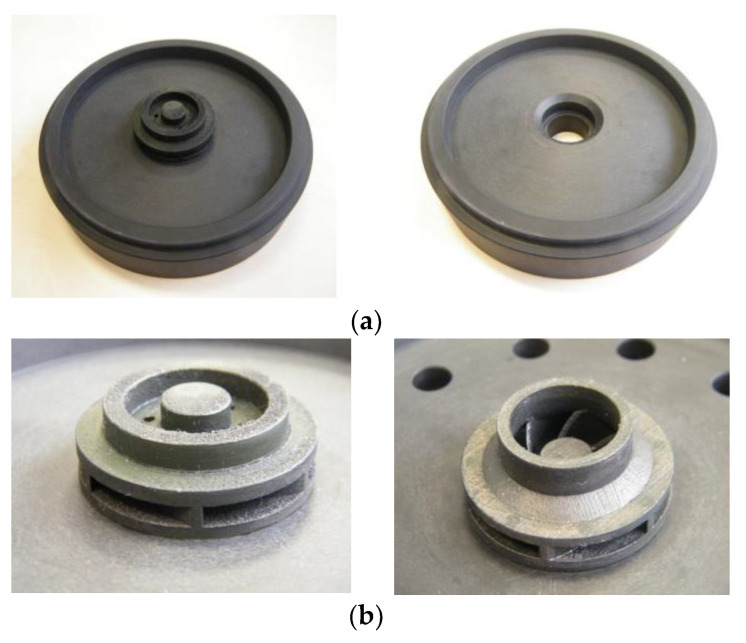
Illustration of the (**a**) customized tool required for performing the AFM finishing process and (**b**) the closed impeller mounted on the customized tool.

A preliminary AFM process was investigated in a previous study [22], showing that the finishing process could be applied to the AM closed impeller, with such small dimensions. However, the process required different optimization approaches, such as the use of optimized tooling, and different media with higher viscosity, pressure and number of cycles. For the current study, the post-processing activities were performed on two closed impellers. Special tooling was required to aid the orientation of the abrasive media, as presented in Figure 8. AFM process parameters are presented in Table 4. The dark-grey color of the closed impeller is due to heat treatment.

**Figure 9:**
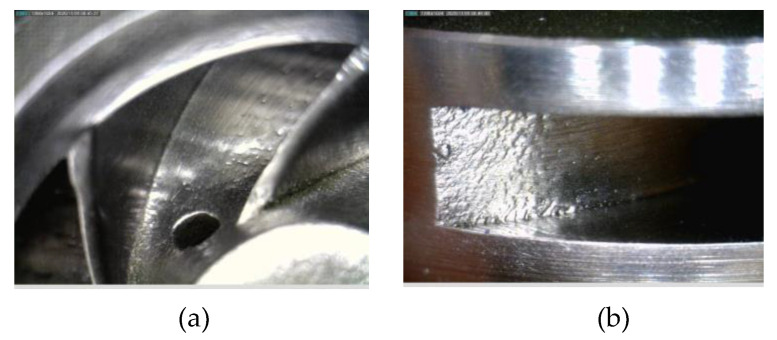
Details of first impeller (after tooling optimization) showing (**a**) interior surface and (**b**) pressure side.

**Figure 10:**
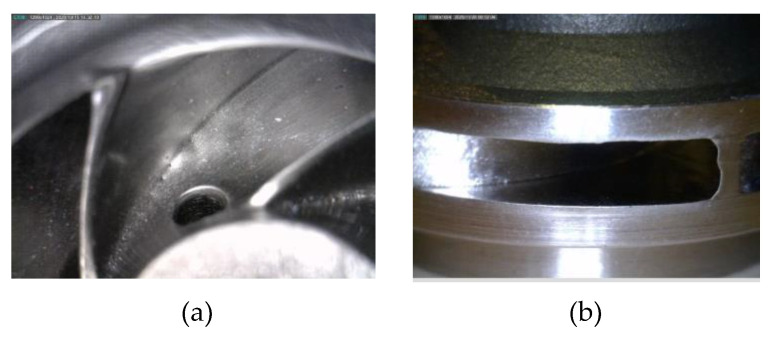
Details of Impeller 2 (after media change) showing (**a**) interior surface and (**b**) pressure side.

After a visual inspection performed on the first closed impeller, the AFM finishing process showed good results in terms of surface roughness improvement. Nevertheless, small areas at the suction side of each blade tail remained unfinished. This resulted from the impeller blades’ geometry, which does not guide the media along with the full extent of the blade. Details of the internal surface area after the AFM process are presented in Figure 9. The second impeller presents a more uniform surface, including the suction side of each blade, and the dimensions (edges and channels) are less affected. Details of the second impeller subjected to the AFM process are presented in Figure 10. 

**Figure 11:**
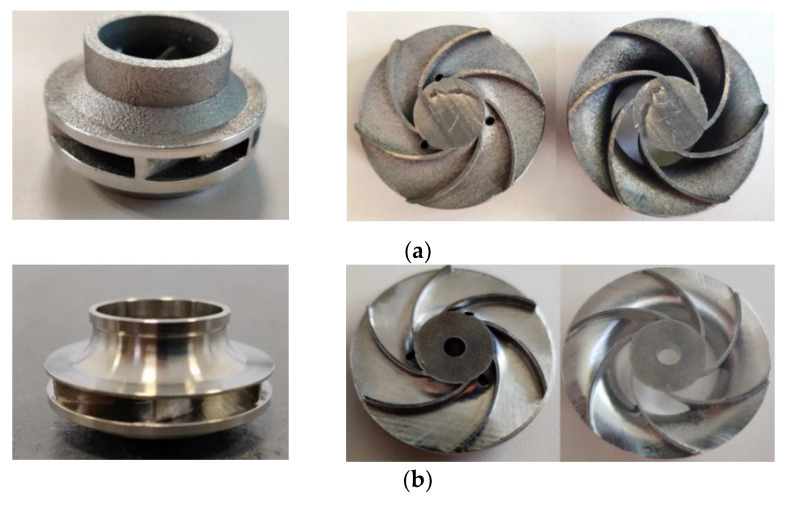
AM closed impeller: (**a**) before post-processing (no heat treatment applied); (**b**) after post-processing all surfaces.

After the AFM process, the closed impeller was mechanically post-processed by means of turning operation, to evaluate the dimensional stability of the outer surfaces. A comparison between the as-printed closed impeller and finished one is presented in Figure 11.

Both impellers were halved (Figure 11) in order to better observe and analyze the interior finishing, as well as the blade thickness. It should be noted that a total of 2.7 and 2.96 g of material was removed during the first and second AFM processes, respectively (average mass of the closed impeller after heat treatment and removal of support material is of 122 g).

**Figure 12:**
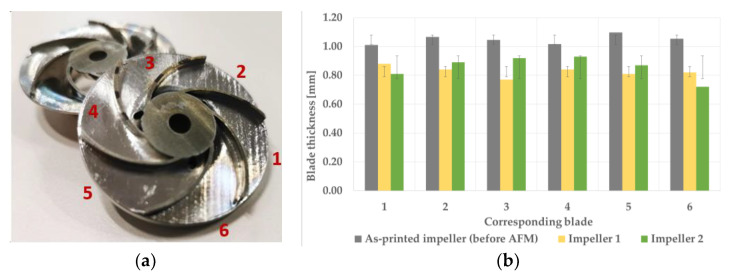
Blade thickness evaluation after AFM process: (**a**) halved AM closed impeller; (**b**) blade thickness values measured using a calibrated equipment with an average of three values with respect to as-printed impeller.

**Figure 13:**
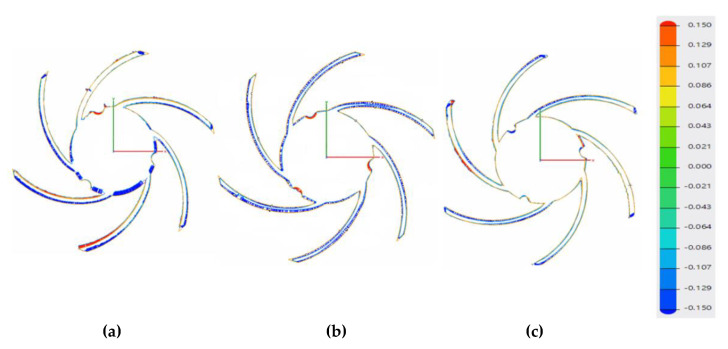
Blade thickness evaluation using 3D measurements for: (**a**) as-printed impeller (before AFM), (**b**) Impeller 1, and **(c)** Impeller 2.

As can be observed in Figure 12 and Figure 13 after the printing process, all six blades’ thicknesses were under the imposed tolerances of ±0.1 mm. As the AFM process affects the blade thickness, both the media and the parameters were modified, according to Table 4. After the second trial, the closed impeller showed better results in terms of blade thickness (Table 5). The media gliding on the blade’s profile induces the differences of thicknesses when compared to the imposed tolerances. 

A roughness evaluation (Table 6) was also performed for the two finished halves of the impeller, and a mean value for the shroud was found at Ra 3.81 μm and for the disc at Ra 0.87 μm, as compared to as-printed values of Ra 7.86 μm for the shroud and Ra 8.13 μm for the disc.

However, in order to evaluate how this affects the overall performances of the pump, a comparison is foreseen by the authors among a conventional manufactured closed impeller, an as-printed closed impeller (mechanically post-process on the outer surfaces), and a fully post-processed AM closed impeller. For this reason, the balancing study was performed on the AM closed impeller only on the exterior surfaces. The third closed impeller was machined to its final dimensions and prepared for Liquid Penetrant Inspection and balancing operations. After performing the LP test on the machined external surfaces, it was found that the closed impeller does not present any visible defects. 

**Figure 14:**
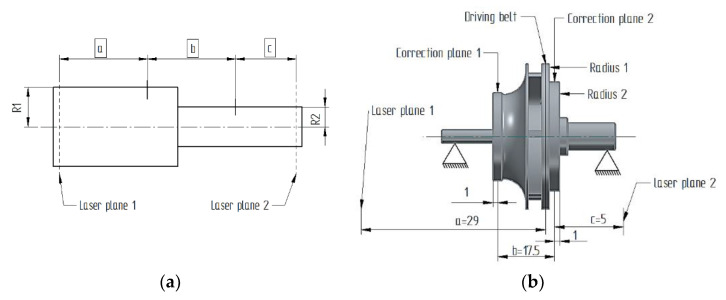
Positioning of the closed impeller on the balancing machine: (**a**) correction plane positioning with corresponding radii; (**b**) closed impeller and shaft mounted on balancing machine.

**Figure 15:**
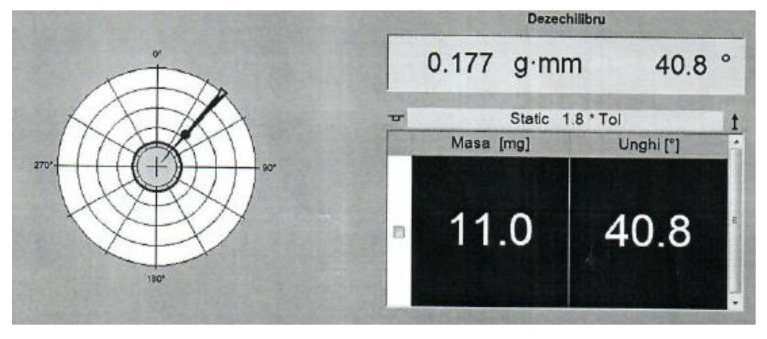
Unbalanced results after balancing operation.

**Figure 16:**
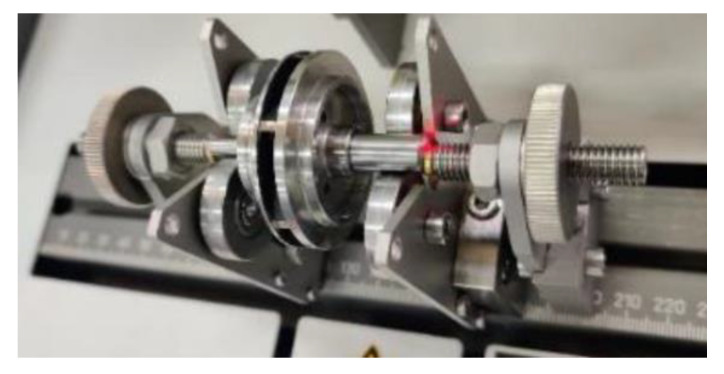
Closed impeller mounted on the balancing machine.

The balancing was performed considering a one-correction plane analysis to veri-fy if the AM closed impeller could be balanced, taking into consideration the possible geometrical deviations that may occur due to the manufacturing and post-processing operations. The closed impeller after being fitted to the dynamic balancing machine is rotated on high speed (2200 rot/min) in order to determine any possible unbalances. The positioning of the closed impeller with the shaft of the balancing machine is pre-sented in Figure 14. The analysis presented in Figure 15 was recorded during the bal-ancing procedure. As mentioned previously, it should be noted that the closed impeller subjected to this balancing trial was not exposed to AFM surface finishing post-processing, as the outcome of this trial was to validate the balancing procedure for such small additively manufactured closed impellers, using only a correction plane. The closed impeller mounted on the balancing machine is illustrated in Figure 16. 

After the first balancing procedure, a mass of 72.8 mg was removed at the corresponding 90° angle, after which the closed impeller was retested, concluding that a secondary mass removal (results presented in Figure 16) is not necessary as the unbalance is within the tolerance defined by the G2.5 balancing class (0.1 g·mm). The balancing results are presented in Table 7. After mass removal, it was concluded, based on the obtained results, that the AM closed impeller can be balanced using a single correction plane.

## 4. Discussion

The high interest in the space industry for the MPFL systems is reflected in the development processes of individual components, where the additive manufacturing was successfully integrated. The main objective of the present paper was to define a complete manufacturing process for a closed impeller by means of LPBF technology, with respect to dimensions accuracy and surface quality. The geometry of the closed impeller presented a challenge for the LPBF technology with respect to the deposition of support material in an unreachable area. 

As the closed impeller is designed for a centrifugal pump that shall be further integrated in MPFL systems, the dimensional accuracy and roughness of the internal surfaces of the closed impeller have a major impact on the pump’s lifetime. Therefore, the current research study focused on presenting an evaluation process for a closed impeller in terms of geometrical and dimensional stability, post-processing activities and a balancing activity for such small AM rotary parts. More precisely, with respect to previous investigations on the manufacturing process of small closed impellers [22,33], the present paper started with the closed impellers manufactured using B + 60° as the building orientation with a laser power of 200W in order to avoid any defects, such as adherent dross on the shroud, being a self-supporting structure.

Before the 3D measurements of closed impellers, the support material was removed by a turning operation. Figure 6 presents the dimensional accuracy for the as-printed part, highlighting the dimensional accuracy of the printing process (geometrical constrains of ±0.1 mm on the blades positioning and tolerances), with respect to the CAD model. Considering the offset material, the geometrical deviations from the back of the closed impeller are not considered, as the turning process removed between 0.1 and 0.2 mm of material during support structure removal. 

The finishing AFM process was investigated using a new type of medium and adjusting the process parameters. The optimization process focused on reducing the impact over the dimensions of the closed impeller. Consistent results were achieved regarding the roughness of the internal surface area. Compared with [22] where the roughness after AFM process for the shroud was found at Ra 3.85 μm and for the disc at Ra 0.66 μm, after optimization approaches applied in the current study, the roughness for the shroud was improved (Ra 2.7 μm), and it was slightly increased for the disc Ra 0.9 μm; however, a better protection was found for the blade geometry of the closed impeller after process optimization. 

The balancing investigation of the closed impeller aimed to achieve a dynamically balanced rotary component that, when installed on the MPFL system, induces an acceptable magnitude of vibration. 

This paper presents not only the advantages of LPBF technology, but also the challenges of the manufacturing process, in this case, the surface quality. Depending on the applicability of the AM component, a compromise was made during the manufacturing process between surface quality and dimensional accuracy. Further investigations on the AM closed impeller will be conducted with a focus on the efficiency of the AM part compared to the cast or welded counterpart.

## 5. Conclusions

This study represents a new step in understanding the complexity of additive manufacturing technology applied for the design of metallic components for space applications to increase the technological readiness level. In addition, a customized post-processing method for the interior finishing of complex geometries, such as closed components, was studied and presented. 

Within this study, a full fabrication process of an Inconel 625 closed impeller for MPFL systems was investigated, by means of LPBF technology. The AM closed impeller was built at B + 60° orientation. X-ray CT scans were conducted to analyze possible defects that may occur during fabrication (porosity, cavitation, voids, inclusions, etc.), showing a very small void content that did not affect the material properties or the performance of the closed impeller. Post-processing operations showed good results in terms of roughness and dimensional stability; however, the AFM process could be further enhanced by using more adaptable abrasive media and process parameters in order to achieve a homogenous finishing process over the blades. 

A balancing study was performed on the closed impeller at a balancing class of G2.5 using a single correction plane, bringing us one step closer to integrating the AM closed impeller into the MPFL system and testing its performance under relevant conditions, in comparison to a conventionally made closed impeller. 

## Figures and Tables

**Table 1:** Chemical composition of metal powder.

Chemical Comp.	Al	C	Co	Cr	Fe	Mo	Nb	Si	Ti	Ni
Lot (%wt)	0.09	0.02	<0.1	21.2	4	9	3.62	<0.05	<0.05	Bal.

**Table 2:** LPBF process parameters [33].

Building Orientation	Laser Power (W)	Scanning Speed (mm/s)	Layer Thickness (µm)	Hatch Distance (mm)	Laser Focus (µm)
B + 60°	200	750	50	0.11	70–120

**Table 3:** Tensile test results of 3 mm gauge diameter specimens machined from 3.5 mm diameter printed.

Specimen No.	Specimen 1	Specimen 2	Specimen 3
d_0_ [mm]	2.984	3.014	2.982
Rm [MPa]	776	769	769
Rp_0.2_ [MPa]	472	465	465
RA [%]	51	51	50
EL [%]	50	50	50

where d0—measured diameter of the test specimen; Rm—e tensile strength of the specimen; Rp0.2—yield strength; RA—reduction in area; EL—elongation.

**Table 4:** AFM process parameters.

Trial	Media Type	Pressure [bar]	Volume [m^3^]	No. of Cycles
Closed Impeller 1 (tooling change)	649 Z1 BC	35	0.00819	10
Closed Impeller 2	EM 25048	50	0.00819	8

**Table 5:** Average blade thickness after AFM finishing process.

	Blade 1 [mm]	Blade 2 [mm]	Blade 3 [mm]	Blade 4 [mm]	Blade 5 [mm]	Blade 6 [mm]
As-printed	1.01	1.07	1.05	1.02	1.10	1.05
Std. dev.	0.06	0.04	0.03	0.06	0.04	0.09
Impeller 1	0.88	0.84	0.77	0.84	0.81	0.82
Std. dev.	0.017	0.062	0.045	0.04	0.065	0.075
Impeller 2	0.81	0.89	0.92	0.93	0.87	0.72
Std. dev.	0.06	0.10	0.01	0.06	0.03	0.03

**Table 6:** Roughness evaluation.

Average Values	Disc		Shroud	
	Ra [µm]	Rz [µm]	Ra [µm]	Rz [µm]
As-printed impeller	7.8681	60.9075	8.138	45.898
Std. dev.	1.019	9.686	0.784	3.478
Impeller 1	0.532	3.843	3.813	33.573
Std. dev.	0.093	0.491	1.602	7.280
Impeller 2	0.907	6.076	2.758	30.085
Std. dev.	0.234	1.598	1.546	13.619

**Table 7:** Closed impeller balancing test.

**Measuring speed for first test**	2231 rot/min	
**Correction plane**	1.09 g·mm	90.0°
**Correction plane—Mass removal**	72.8 mg	90.0°
**Measuring speed for second test**	2208 rot/min	
**Correction plane**	0.177 g·mm	40.8°
**Correction plane—Mass removal**	11.0 mg	40.8°

## Data Availability

The data presented in this study are available on request from the corresponding author. Due to the contract agreement between COMOTI and the funding agency, the research activities and data presented in this paper will be presented in an Executive Summary Report and will be available for public use, after project closure.
